# Outer Membrane Vesicles of Vibrio cholerae Protect and Deliver Active Cholera Toxin to Host Cells via Porin-Dependent Uptake

**DOI:** 10.1128/mBio.00534-21

**Published:** 2021-05-26

**Authors:** Franz G. Zingl, Himadri B. Thapa, Martina Scharf, Paul Kohl, Anna M. Müller, Stefan Schild

**Affiliations:** a Institute of Molecular Biosciences, University of Graz, Graz, Austria; b BioTechMed Graz, Graz, Austria; c Field of Excellence Biohealth, University of Graz, Graz, Austria; Institut Pasteur

**Keywords:** OmpT, OmpU, *Vibrio cholerae*, cargo, cholera toxin, gastrointestinal tract, human intestinal cells, internalization, outer membrane vesicles, rhodamine, trypsin, virulence

## Abstract

Outer membrane vesicles (OMVs) are an emerging research field due to their multifactorial composition and involvement in interspecies and intraspecies communication. Recent studies indicate that vesicle release by Gram-negative bacterial pathogens is increased during *in vivo* colonization, as exemplified by the facultative human pathogen Vibrio cholerae upon oral ingestion by the host. In this study, we investigate the fate of OMVs produced by the Gram-negative facultative pathogen V. cholerae. We show that vesicles produced by the clinically relevant El Tor biotype are readily taken up by human intestinal cell lines. We identify outer membrane porins of V. cholerae, i.e., OmpU and OmpT, as the required surface effectors on OMVs for cellular uptake, and we pinpoint the uptake mechanism as caveolin-mediated endocytosis. Furthermore, we show that OMVs derived from V. cholerae grown under virulence-inducing conditions act as potent vehicles for delivery of bioactive cholera toxin to intestinal epithelial cells. In contrast to free cholera toxin secreted via the type II secretion system, OMV-associated cholera toxin is protected from degradation by intestinal proteases. Taken together, these data show that OMV-associated cholera toxin can sustain longer periods in the intestinal tract and preserve toxin effects, as indicated by a prolonged increase of cAMP levels in the intestinal tissue.

## INTRODUCTION

Gram-negative bacteria naturally secrete nanometer-scale, spherical particles from their outer membrane, commonly referred to as outer membrane vesicles (OMVs) (1–3). These multifactorial complexes range in diameter from 10 to 300 nm and consist mainly of microbial surface components, such as phospholipids, (lipo)proteins, outer membrane proteins, peptidoglycan, lipopolysaccharides (LPS), or lipooligosaccharides. OMVs can also contain periplasmic components, which are trapped in the lumen of the OMVs during the vesiculation process. OMVs have been ascribed diverse physiological functions, including roles in nutrient acquisition due to the presence of degradation enzymes (e.g., proteases), resistance to bacterial surface-attacking antimicrobial agents (e.g., complement system or phages resistance), and intraspecies and interspecies communication mediated by OMV-containing signaling molecules (e.g., quorum-sensing autoinducers or small RNAs [sRNAs]) (1). A recent study extended the pathophysiological roles of OMVs, demonstrating that they contribute to surface remodeling of the facultative human pathogen Vibrio cholerae along the environment to *in vivo* transition (4).

OMVs released by Gram-negative pathogens can serve as delivery vehicles for immunomodulatory effectors and toxins or as a sink for host-derived antimicrobial factors, such as defensins and the complement system (3, 5, 6). Indeed, current data suggest that Gram-negative bacteria exhibit increased OMV release during host colonization, which is triggered by *in vivo* stressors such as iron limitation and envelope stress (4, 6, 7). The increased vesiculation upon host entry promotes *in vivo* adaptation of the facultative pathogen V. cholerae to antimicrobial intestinal stressors via enhanced surface exchange (4). This includes the accumulation of (di)glycine-modified LPS and the removal of OmpT, resulting in greater resistance to cationic antimicrobial peptides and bile, respectively (8). OMVs of V. cholerae have also been shown to alter the inflammatory response of epithelial cells and to act as a sink to counteract bacteriophage infections (9–12). Moreover, they represent promising vaccine candidates because they induce a robust protective immune response (9–12).

Notably, V. cholerae is the causative agent of the severe human diarrheal disease cholera, with an overall global burden estimated to be 3 to 5 million cases and up to 130,000 deaths per year (13). Infection usually starts with the oral ingestion of V. cholerae through contaminated food or water (13–15). After passage through the stomach, V. cholerae reaches the small intestine, its primary site of colonization, and induces expression of virulence factors such as the toxin-coregulated pilus (TCP) and cholera toxin (CT) (13–16). The expression of virulence factors is controlled by a complex regulatory cascade, also known as the ToxR regulon (17). It includes the membrane-bound protein complexes ToxR-ToxS and TcpP-TcpH and the cytosolic ToxT, a member of the AraC family of transcriptional regulators. Most of the virulence factors, including CT and TCP, are regulated by the ToxT-dependent pathway of the ToxR regulon in response to intestinal stimuli (18). ToxR-ToxS and TcpP-TcpH act in concert to activate expression of ToxT, which subsequently induces CT and TCP expression (19). ToxR can also directly regulate several genes independent of ToxT. For example, ToxR activates OmpU and silences OmpT porin expression, which plays an essential role in achieving bile resistance and full virulence (12, 20).

As a classic A-B-type toxin, the CT holotoxin is composed of one CT-A subunit and five CT-B subunits. After Sec-dependent secretion of the individual subunits, correct folding occurs in the periplasm before the assembled CT is translocated via the type II secretion system (T2SS) across the outer membrane into the extracellular milieu (21, 22). The pentameric ring structure of CT-B ensures binding to host cells via the GM1 ganglioside receptor (23, 24), while CT-A catalyzes the ADP-ribosylation of the stimulatory G-protein Gsα. This results in constitutive activation of adenylate cyclase and thereby gives rise to elevated levels of cAMP within the host cell (25, 26). This increases chloride and bicarbonate secretion by intestinal cells, while sodium uptake is reduced. Along the ion gradient, water passively leaks into the intestinal lumen, which manifests in severe secretory diarrhea.

Importantly, the presence of CT in OMVs has been recently reported for classical V. cholerae strains, i.e., the 596B isolate with relatively high CT production (27, 28). While one report suggests GM1-dependent uptake of OMV-associated CT from classical V. cholerae, another study indicates GM1-independent cell entry of OMV-associated CT, especially if OMVs are isolated from classical V. cholerae grown under low-salt conditions (27, 29). Thus, information on the relevant effectors on the bacterial or host cell surface is currently lacking or inconclusive. Moreover, the classical biotype is thought to be extinct and replaced by isolates of the El Tor biotype, which have been responsible for all major cholera outbreaks within the past 60 years (15, 26). Although the classical and El Tor biotypes belong to the O1 serogroup, they differ by phenotypic features, highlighted by differential virulence factor expression (30, 31). While the classical 596B strain shows already high CT production upon growth at 30°C in liquid medium with low pH (∼6.5) and osmolarity (∼66 mM), El Tor strains exhibit a more refined virulence regulation, requiring a defined liquid broth called AKI with microaerophilic cultivation followed by intense aeration to induce virulence (29, 32). Association of CT with OMVs in V. cholerae El Tor under virulence-inducing AKI conditions has so far not been investigated.

Based on the elevated OMV production of V. cholerae El Tor upon host entry, we aimed to study the subsequent fate of the OMVs and their potential interactions with host cells in more detail. We identify the bacterial surface factors relevant for OMV uptake by human intestinal cells and elucidate the cellular uptake pathway. Finally, we provide a pathophysiological function, confirming that OMVs derived from V. cholerae El Tor contain active CT, which, in contrast to freely secreted CT, is protected from degradation by the intestinal protease trypsin.

## RESULTS

### V. cholerae OMVs are readily taken up by human intestinal cells.

**FIG 1:**
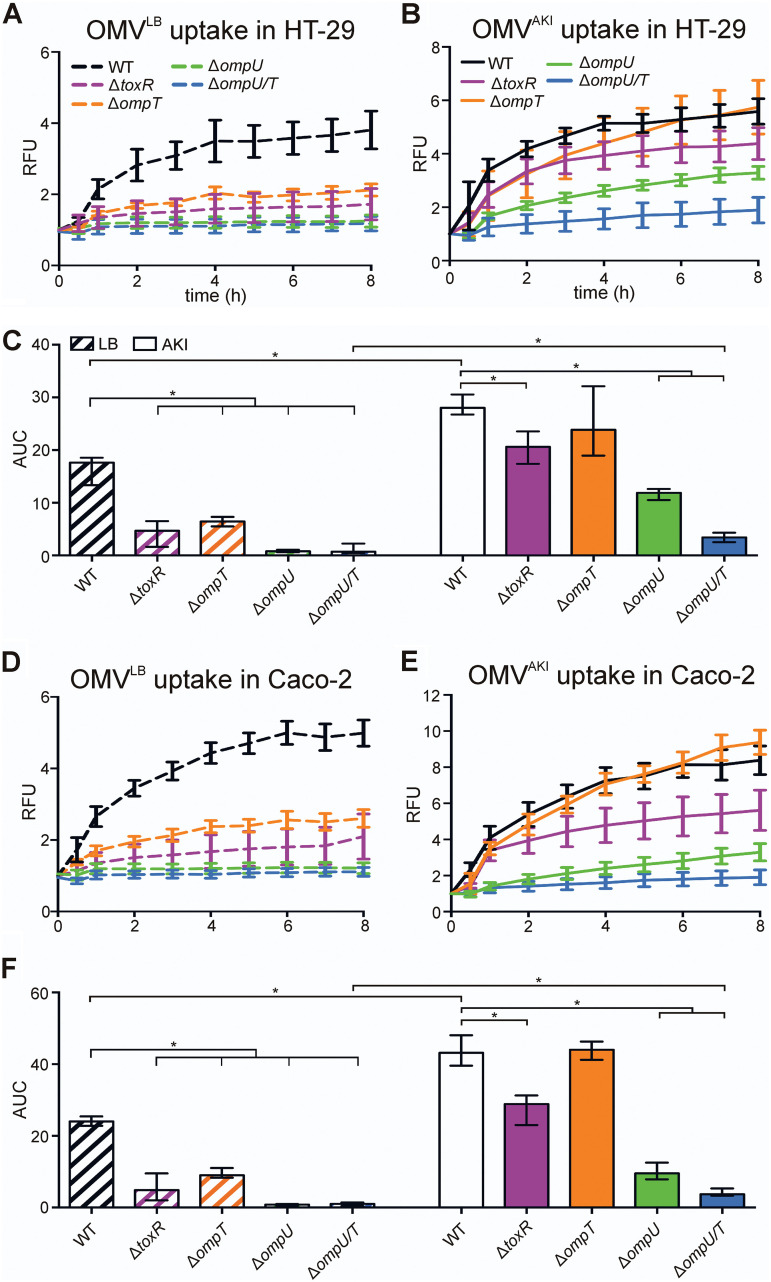
Intestinal epithelial cell uptake of OMVs derived from V. cholerae depends on outer membrane porins OmpU and OmpT. (A, B, D, and E) Intestinal epithelial HT-29 cells (A and B) or Caco-2 cells (D and E) were incubated for 8 h with rhodamine-labeled OMVs derived from WT V. cholerae or Δ*toxR*, Δ*ompT*, Δ*ompU*, or Δ*ompU*/*T* mutants grown in LB (A and D) or under virulence-inducing conditions using AKI (B and E). Uptake was detected by an increase in relative fluorescence units (RFU) measured every 1 h. Wells containing rhodamine-labeled OMVs without cells served as a blank. Shown are the mean ± standard deviation (SD) (*n* ≥ 8). (C and F) Shown are the median AUC values ± interquartile range (IQR) retrieved from the uptake analyses in HT-29 cells in panels A and B (C) and in Caco-2 cells in panels D and E (F). Asterisks highlight significant differences between respective data sets. *, *P < *0.05, Kruskal-Wallis test followed by *post hoc* Dunn’s multiple comparisons.

To assess OMV uptake dynamics of V. cholerae OMVs in human intestinal epithelial cell lines, we used a fluorescence measurement in combination with rhodamine R18-labeled OMVs, as established previously for vesicles derived from other Gram-negative bacteria (33, 34). Due to high rhodamine R18 concentrations in OMVs, the fluorescence signal is quenched, but fluorescence intensity increases as the rhodamine probe is diluted by fusion of the OMV with the host membrane. Density gradient-purified rhodamine R18-labeled OMVs from V. cholerae cultivated under non-virulence-inducing conditions (i.e., lysogeny broth [LB]) and under virulence-inducing conditions (i.e., AKI) were incubated with the human intestinal cells, and fluorescence intensity was quantified every 1 h over an 8-h period. Independent of the cultivation condition, OMVs from wild-type (WT) V. cholerae are readily taken up by HT-29 cells, reaching maximum levels within 4 h (Fig. 1A and B). Assays using OMVs derived from AKI-grown WT V. cholerae reached higher fluorescent maxima, compared to LB-grown OMVs, indicating better uptake efficacy. Accordingly, the area under the curve (AUC), reflecting the overall OMV uptake within the observed time, was significantly higher for WT OMVs derived from AKI cultures (Fig. 1C).

### Uptake of OMVs depends on outer membrane porins.

To elucidate the relevant bacterial surface factors for host cell internalization, we compared the internalization by HT-29 cells using OMVs from diverse V. cholerae surface mutants cultivated in LB and AKI (Fig. 1; also see Fig. S1 in the supplemental material). These included the LPS Δ*rfbA*-*T* mutant, lacking the O antigen, and the deep rough Δ*wav* mutant, lacking a substantial part of the core oligosaccharide in addition to the O antigen. Furthermore, OMVs derived from the flagellum biosynthesis Δ*flrA* mutant and outer membrane porin Δ*ompU*, Δ*ompT*, and Δ*ompU*/*T* mutants were analyzed. Finally, OMVs from regulator Δ*toxR* and Δ*tcpP* mutants, which are impaired in outer membrane porin regulation and/or virulence gene activation, were tested. Among all OMVs tested, only those derived from outer membrane porin Δ*ompU*, Δ*ompT*, and Δ*ompU*/*T* mutants and the regulator Δ*toxR* mutant showed reduced uptake dynamics (Fig. 1A to C). Notably, Δ*toxR* mutants exhibit high OmpT levels but almost no OmpU on the bacterial surface (12, 35). Thus, altered abundance of outer membrane proteins OmpU and OmpT affects OMV uptake in HT-29 cells. In detail, under non-virulence-inducing conditions (LB), uptake of OMVs from Δ*ompT* and Δ*toxR* mutants is significantly reduced, while internalization of OMVs from Δ*ompU* and *ompU*/*T* mutants is almost abolished (Fig. 1A and C). Thus, OMV uptake of LB-derived OMVs mainly depends on OmpU.

10.1128/mBio.00534-21.1 Fig S1 Intestinal epithelial cell uptake of OMVs derived from WT V. cholerae and diverse surface mutants. (A and C) HT-29 intestinal cells were incubated for 8 h with rhodamine-labeled OMVs derived from WT V. cholerae as well as Δ*rfbA*-*T*, Δ*wav*, Δ*tcpP*, and Δ*flrA* mutants grown in LB (A) or AKI (C). Uptake is detected by an increase in RFU measured every 1 h. Wells containing rhodamine-labeled OMVs without cells served as a blank. Shown is the mean ± SD (*n* ≥ 8). (B and D) Shown are the median AUC values ± IQR retrieved from the uptake analyses of LB (B) or AKI (D) values. Download FIG S1, PDF file, 0.3 MB. Copyright © 2021 Zingl et al. 2021 Zingl et al. https://creativecommons.org/licenses/by/4.0/ This content is distributed under the terms of the Creative Commons Attribution 4.0 International license.

Based on the enhanced uptake of WT V. cholerae-derived OMVs (Fig. 1A and B) and to better reflect *in vivo* scenarios, we also analyzed the uptake of mutant OMVs derived from AKI cultures, which induce the ToxR regulon, resulting in *ompU* activation and *ompT* repression (36). Consistent with observations under non-virulence-inducing conditions, OMVs from Δ*ompU* cultivated in AKI showed a significant reduction in uptake, which was even more pronounced for OMVs from the Δ*ompU*/*T* double mutant (Fig. 1B). However, OMVs from the Δ*ompT* mutant cultivated in AKI showed similar uptake dynamics, compared to WT OMVs (Fig. 1B). This suggests that OmpU also has a dominant role in host cell uptake of OMVs derived under virulence-inducing conditions, while additional loss of OmpT further reduces uptake to minimal levels. In summary, OMVs derived from a Δ*ompU*/*T* double mutant exhibit the least uptake under LB and AKI conditions. Concordant with uptake studies with OMVs derived from LB cultures, AKI culture-derived OMVs from LPS mutants (Δ*rfbA*-*T* and Δ*wav* mutants), from the flagellum Δ*flrA* mutant, or from the regulator Δ*tcpP* mutant demonstrated similar internalization dynamics, compared with WT OMVs (Fig. S1). Thus, cellular uptake of V. cholerae OMVs does not depend on LPS, flagellar components, or any component regulated via the ToxT-dependent virulence cascade, such as TCP.

The internalization assays described above indicate that OMVs derived from a Δ*ompU*/*T* double mutant exhibit the lowest uptake into HT-29 cells. In *trans* expression of both OmpU and OmpT in the Δ*ompU*/*T* double mutant significantly enhanced uptake of the OMVs, in comparison to OMVs derived from the Δ*ompU*/*T* double mutant carrying an empty vector (Fig. S2). Notably, no significant difference was observed for OMVs derived from the Δ*ompU*/*T* mutant expressing OmpU or OmpT, indicating that the two porins can restore the uptake in HT-29 cells to similar levels. It should be emphasized that expression from a plasmid using the same promoter yields similar protein levels, whereas OmpU is more abundant than OmpT in WT V. cholerae with chromosomal expression from their natural promoters. This is already observable under non-virulence-inducing LB conditions but becomes even more pronounced upon virulence induction in AKI, due to activation of the ToxR cascade (35). Thus, it seems that both porins can mediate uptake in intestinal cells but OmpU is the dominant factor, due its greater abundance in WT OMVs. Concordantly, OMVs derived from the Δ*toxR* mutant exhibit only slightly reduced uptake in HT-29 cells, compared to WT OMVs, due to high OmpT levels compensating for the loss of OmpU. Any impact by a factor of the ToxT-dependent virulence regulon can be excluded because LB- and AKI-grown OMVs from Δ*tcpP* show similar uptake, compared to the respective WT OMVs (Fig. S1).

10.1128/mBio.00534-21.2 FIG S2 Expression of OmpU and OmpT in *trans* restores OMV uptake in intestinal epithelial cells. (A) HT-29 intestinal cells were incubated for 8 h with rhodamine-labeled OMVs derived from V. cholerae Δ*ompU*/*T* p, Δ*ompU*/*T* pOmpU, and Δ*ompU*/*T* pOmpT grown in AKI. Uptake is detected by an increase in RFU measured every 1 h. Wells containing rhodamine-labeled OMVs without cells served as a blank. Shown is the mean ± SD (*n* ≥ 8). (B) Shown are the median AUC values ± IQR retrieved from the uptake analyses presented in panel A. Asterisks highlight significant differences between respective data sets. *, *P < *0.05, Kruskal-Wallis test followed by *post hoc* Dunn’s multiple comparisons. Download FIG S2, PDF file, 0.2 MB. Copyright © 2021 Zingl et al. 2021 Zingl et al. https://creativecommons.org/licenses/by/4.0/ This content is distributed under the terms of the Creative Commons Attribution 4.0 International license.

Uptake assays were also performed in a second human intestinal cell line, i.e., Caco-2, to exclude any cell line-specific effects (Fig. 1D to F). Consistent with the results obtained from HT-29 cells, internalization assays with Caco-2 cells demonstrated (i) slightly more effective uptake of OMVs derived under virulence-inducing conditions, (ii) a dominant role of OmpU in host cell uptake, and (iii) the strongest defect in uptake of OMVs derived from a Δ*ompU*/*T* double mutant. Thus, porin-dependent uptake of V. cholerae OMVs seems to be conserved among human intestinal epithelial cells.

For the rest of the study, we focused on OMVs derived under virulence-inducing conditions (AKI) due to their slightly enhanced uptake efficacy, compared to OMVs derived from LB cultures, for both cell lines and closer simulation of the *in vivo* situation during intestinal colonization.

### OMV uptake is blocked by nystatin or dynasore.

**FIG 2:**
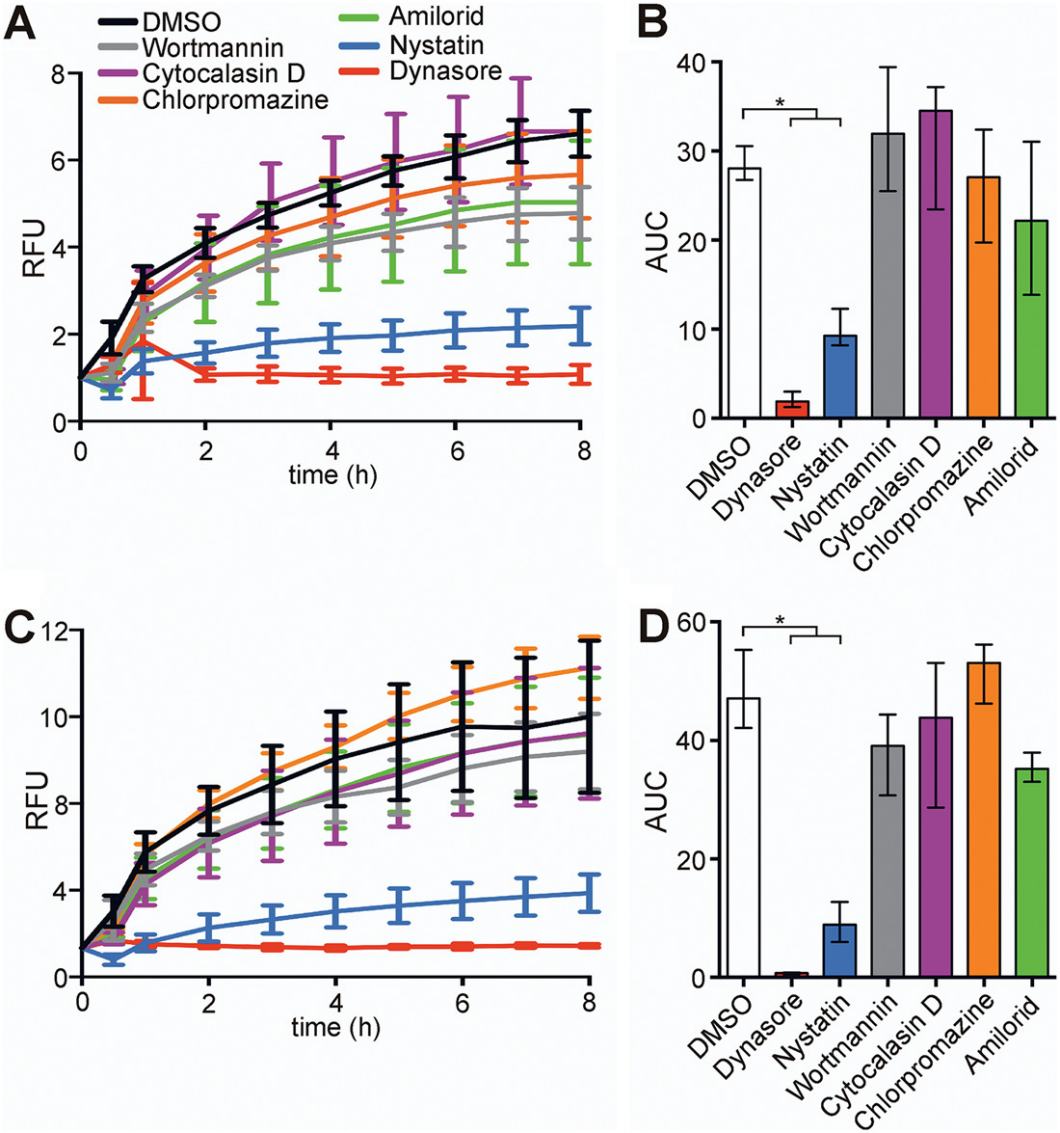
Intestinal epithelial cell uptake of OMVs derived from V. cholerae is significantly reduced by dynasore or nystatin. (A and C) Intestinal epithelial HT-29 cells (A) or Caco-2 cells (C) were incubated for 8 h with rhodamine-labeled OMVs derived from WT V. cholerae in the presence of uptake inhibitors wortmannin, cytochalasin D, chlorpromazine, amiloride, nystatin, or dynasore or the solvent DMSO (control). Uptake is detected by an increase in RFU measured every 1 h. Wells containing rhodamine-labeled OMVs from WT V. cholerae without cells served as a blank. Shown is the mean ± SD (*n* ≥ 6 for panel A and *n* ≥ 4 for panel C). (B and D) Shown are the median AUC values ± IQR retrieved from the uptake analyses in HT-29 cells in panel A (B) and in Caco-2 cells in panel C (D). Asterisks highlight significant differences between respective data sets. *, *P < *0.05, Kruskal-Wallis test followed by *post hoc* Dunn’s multiple comparisons.

**FIG 3:**
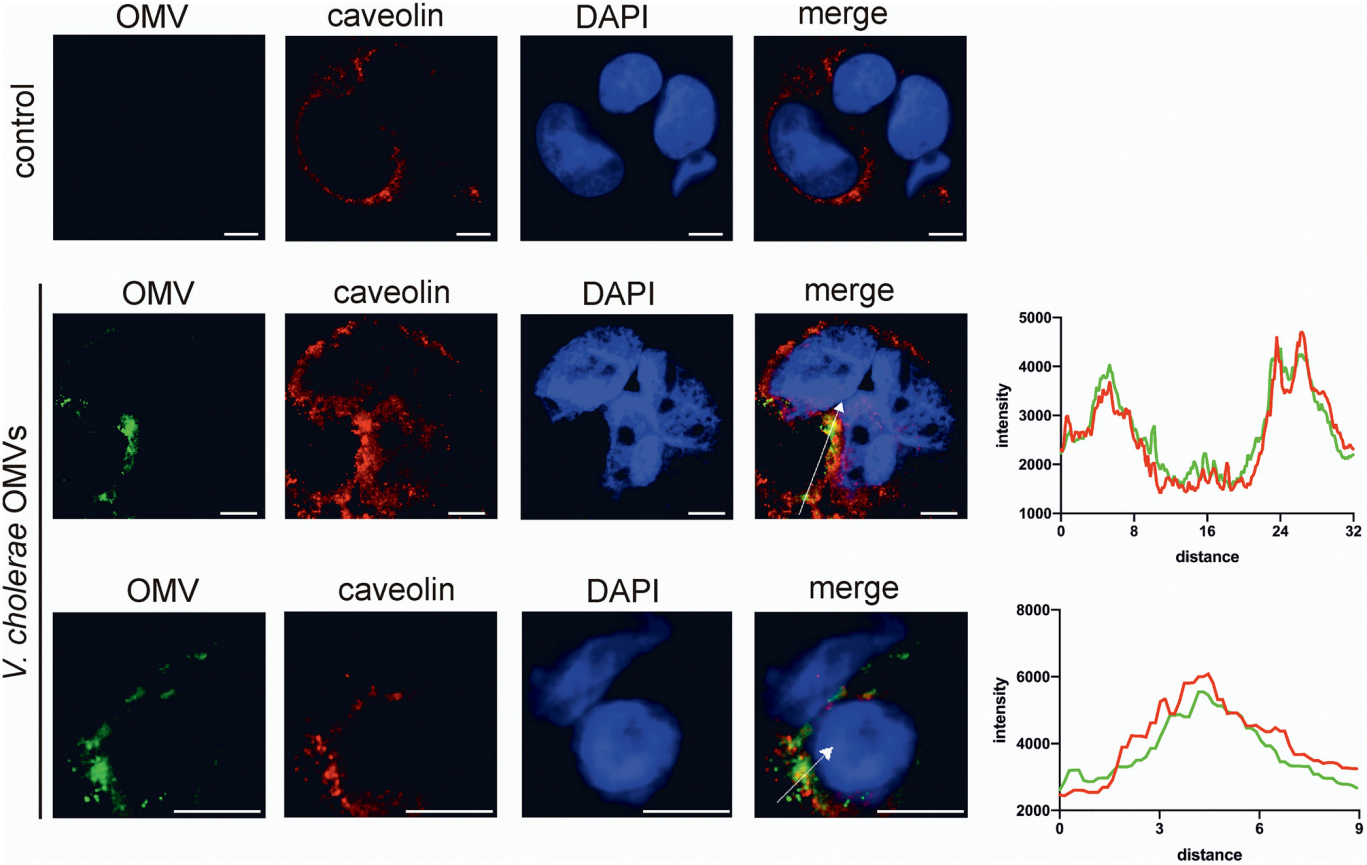
OMVs derived from V. cholerae colocalize with caveolin of intestinal epithelial cells. Shown are representative fluorescent images of Caco-2 cells that were mock treated with saline (control) (top row) or incubated with WT V. cholerae OMVs (middle and bottom rows) for 10 min. V. cholerae OMVs were stained with mouse-derived anti-OMV antiserum (71) and FITC-conjugated goat anti-mouse IgG (green), and caveolin was stained using a rabbit anti-caveolin 1 antibody and Cy3-conjugated goat anti-rabbit IgG (red). Nuclei were stained with DAPI (blue). Colocalized red and green signals appear yellow in the merged images of cells incubated with V. cholerae OMVs (middle and bottom [independent image at higher magnification] rows). Colocalization of the green (OMVs) and red (caveolin) signals was confirmed by histogram analysis of the fluorescence intensities along the white arrow. Scale bars, 10 μm.

Among the few studies investigating OMV uptake by nonprofessional phagocytic host cells, it becomes obvious that several pathways can be involved and are sometimes even utilized in combination (34, 37–40). To decipher which pathways contribute to the entry of OMVs in nonprofessional phagocytic cells, a set of commercially available uptake inhibitors covering the main reported pathways was used in combination with WT OMVs (Fig. 2). Addition of wortmannin (inhibition of phosphatidylinositol kinases, preventing macropinosome closure), cytochalasin D (inhibition of actin polymerization, preventing membrane fusion), chlorpromazine (inhibition of clathrin-coated pit formation, preventing clathrin-dependent endocytosis), or amiloride (inhibition of Na^+^/H^+^ exchange, blocking micropinocytosis) had no significant effect on the WT OMV uptake dynamics in HT-29 or Caco-2 cells. In contrast, addition of nystatin, which intercalates and disrupts cholesterol‐rich membrane domains, affecting caveolin-mediated endocytosis and lipid raft formation, and especially dynasore, which inhibits dynamin GTPase activity, preventing clathrin-dependent and caveolin-mediated endocytosis, resulted in a significant decrease of OMV uptake, compared to the solvent (dimethyl sulfoxide [DMSO])-treated control (Fig. 2A and C). Accordingly, significant reductions in the AUC values were observed in the presence of nystatin or dynasore, in comparison to the solvent controls, for uptake assays using HT-29 and Caco-2 cells (Fig. 2B and D). Combinatory analyses of the inhibitors and their target pathways strongly suggest that V. cholerae OMVs are predominantly taken up by caveolin-mediated endocytosis, which is blocked by nystatin and dynasore. The contribution of this endocytic pathway to V. cholerae OMV uptake was further assessed by visualizing OMVs and caveolin in Caco-2 cells after a 10-min incubation. Using fluorescence microscopy, colocalization of V. cholerae OMVs and caveolin was observed in the merged images as yellow spots, as confirmed by histogram analysis (Fig. 3). Notably, addition of nystatin and dynasore also blocks uptake of WT OMVs derived from LB cultures in both intestinal cell lines (Fig. S3), indicating that caveolin-mediated endocytosis acts as the dominant uptake pathway for OMVs derived under non- virulence-inducing conditions.

10.1128/mBio.00534-21.3 FIG S3 Dynasore or nystatin also reduces uptake of OMVs derived from WT V. cholerae grown in LB. (A and C) Intestinal epithelial HT-29 cells (A) or Caco-2 cells (C) were incubated for 8 h with rhodamine-labeled OMVs derived from WT V. cholerae in the presence of uptake inhibitors nystatin or dynasore or the solvent DMSO (control). Uptake is detected by an increase in RFU measured every 1 h. Wells containing rhodamine-labeled OMV from WT V. cholerae without cells served as a blank. Shown is the mean ± SD (*n* ≥ 8). (B and D) Shown are the median AUC values ± IQR retrieved from the uptake analyses in HT-29 cells in panel A (B) and in Caco-2 cells in panel C (D). Asterisks highlight significant differences between respective data sets. *, *P < *0.05, Kruskal-Wallis test followed by *post hoc* Dunn’s multiple comparisons. Download FIG S3, PDF file, 0.3 MB. Copyright © 2021 Zingl et al. 2021 Zingl et al. https://creativecommons.org/licenses/by/4.0/ This content is distributed under the terms of the Creative Commons Attribution 4.0 International license.

### OMVs deliver bioactive CT to host cells in a porin-dependent manner.

**FIG 4:**
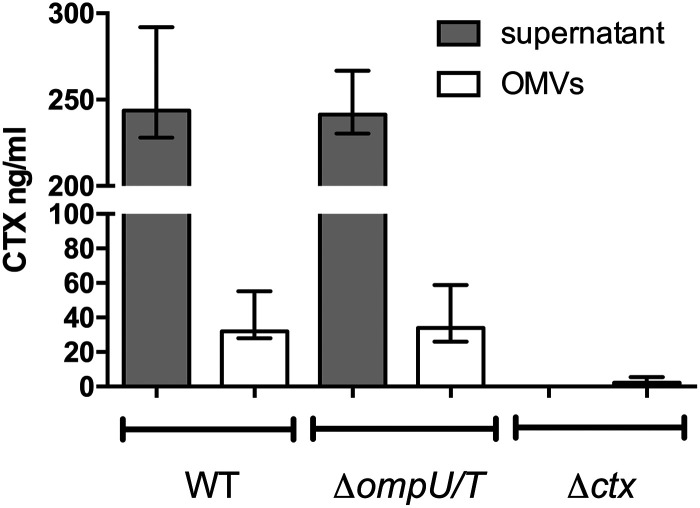
CT is associated with OMVs derived from V. cholerae. The amount of CT in OMVs and supernatants derived from WT, Δ*ompU*/*T*, and Δ*ctx* strains grown under virulence-inducing conditions was quantified by ELISA. Data are presented are median ± IQR (*n* ≥ 6).

The delivery of OMV-associated cargo to bacterial and host cells, promoting intraspecies and interspecies communication, seems to be a relevant physiological role of OMVs (41). Based on reports detecting CT in OMVs from classical strains (27, 28), we quantified by enzyme-linked immunosorbent assay (ELISA) the CT amounts in OMV samples and in the supernatant derived from V. cholerae El Tor grown under virulence-inducing conditions (Fig. 4). Indeed, CT could be detected in the supernatant and in OMVs derived from WT V. cholerae. It should be noted that the filter-sterilized supernatant sample prior to OMV isolation via ultracentrifugation was analyzed. Thus, the supernatant sample comprises T2SS-secreted, soluble CT as well as OMV-associated CT. Based on the ELISA quantification, approximately 10% to 15% of the overall CT is OMV associated. Similar CT levels were obtained for the supernatant and the host cell uptake-deficient OMVs of the Δ*ompU*/*T* double mutant. Supernatant and OMVs derived from the Δ*ctx* mutant served as negative controls for the ELISA, with levels close to or even below the limit of detection. Analyses of vesicle size and biomass quantification revealed no major differences between OMVs derived from WT, Δ*ompU*/*T*, and Δ*ctx* strains (Table 1), suggesting similar vesiculation of all three strains tested.

**TABLE 1:** Qualitative analyses of OMVs derived from AKI cultures of WT, Δ*ompU*/*T*, and Δ*ctx* V. cholerae

Parameter	Data for:		
	WT	Δ*ompU*/*T*	Δ*ctx*
Zetasizer size measurement (nm)			
Mean	119.9 ± 18.16	95.41 ± 24.21	105.5 ± 17.81
Mode	133.2 ± 24.70	108.3 ± 38.53	128.6 ± 16.83
Bradford protein quantification (μg/μl)	2.21 ± 0.8	2.36 ± 0.54	2.99 ± 0.82

**FIG 5:**
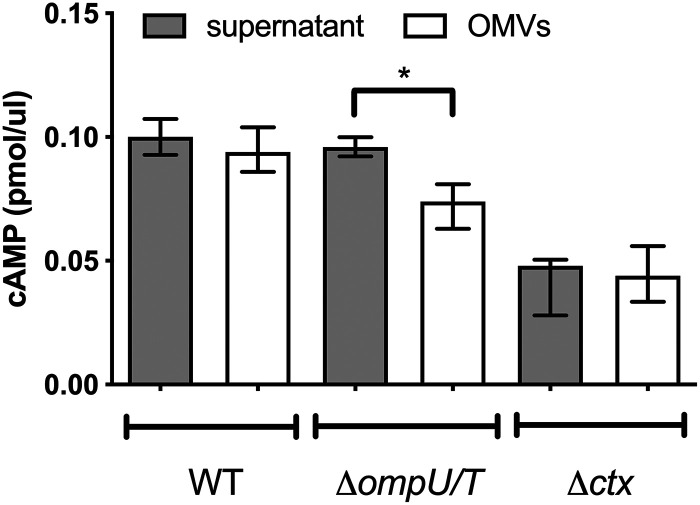
OMV-associated CT increases cAMP levels in intestinal epithelial cells. HT-29 cells were treated with WT, Δ*ompU/T*, and Δ*ctx* strain-derived OMVs and supernatants for 6 h, and the intracellular cAMP levels were assayed to determine the translocation of CT into the host cytosol. Equal CT amounts based on ELISA measurements were used for OMVs and supernatants derived from the WT strain and the Δ*ompU/T* mutant. Due to the absence of CT in samples derived from the Δ*ctx* mutant, equal OMV amounts or supernatant volumes as used for the WT samples were applied. Data are presented as median ± IQR (*n* = 5). Asterisks highlight significant differences between respective data sets. *, *P < *0.05, Kruskal-Wallis test followed by *post hoc* Dunn’s multiple comparisons.

OMVs have been suggested to act as export systems for misfolded proteins (1). Thus, we compared the CT activity by measuring the increase in cAMP levels of HT-29 cells upon exposure to supernatant and OMVs derived from WT V. cholerae grown under virulence-inducing conditions (Fig. 5). The amount of supernatant and OMVs added to the HT-29 cells was adjusted according to the CT quantification via ELISA, to ensure application of equivalent CT levels. Again, supernatant and OMVs derived from the Δ*ctx* mutant served as negative controls and allowed the assessment of basal cellular cAMP levels. Due to the absence of CT in samples derived from the Δ*ctx* mutant, OMV amounts or supernatant volumes equal to those used for the WT samples were applied. Addition of supernatant and OMVs derived from WT V. cholerae resulted in significant increases of cAMP levels, to comparable levels. This indicates that OMV-associated CT is as active as CT present in the supernatant. Consistent with the equal CT amounts quantified by ELISA (Fig. 4), the supernatant of the Δ*ompU*/*T* double mutant resulted in similar cAMP levels, compared with the supernatant derived from the WT strain (Fig. 5). Thus, CT in the supernatants of the WT and Δ*ompU*/*T* strains exhibited similar activity levels. However, addition of OMVs from the Δ*ompU*/*T* double mutant resulted in a significantly smaller cAMP increase, compared to the Δ*ompU*/*T* supernatant or WT samples. Because OMVs of the Δ*ompU*/*T* mutant contain equal amounts of CT, compared to OMVs derived from the WT strain, the most likely explanation for the lower cAMP levels is the impaired uptake of OMVs derived from the Δ*ompU*/*T* mutant.

### OMVs protect CT from degradation in the intestinal milieu.

OMV association of bioactive CT raises the question of its physiological role with regard to free secreted CT. A beneficial effect of OMV packaging is the protection of the cargo from harmful agents (3). Especially the intestinal tract, the primary colonization site of V. cholerae, exhibits relative high concentrations of digestive enzymes, such as the trypsin proteases. Thus, CT secreted by V. cholerae in the intestine might be subject to rapid proteolysis, while OMV-associated CT could be protected from degradation, thereby extending its half-life.

**FIG 6:**
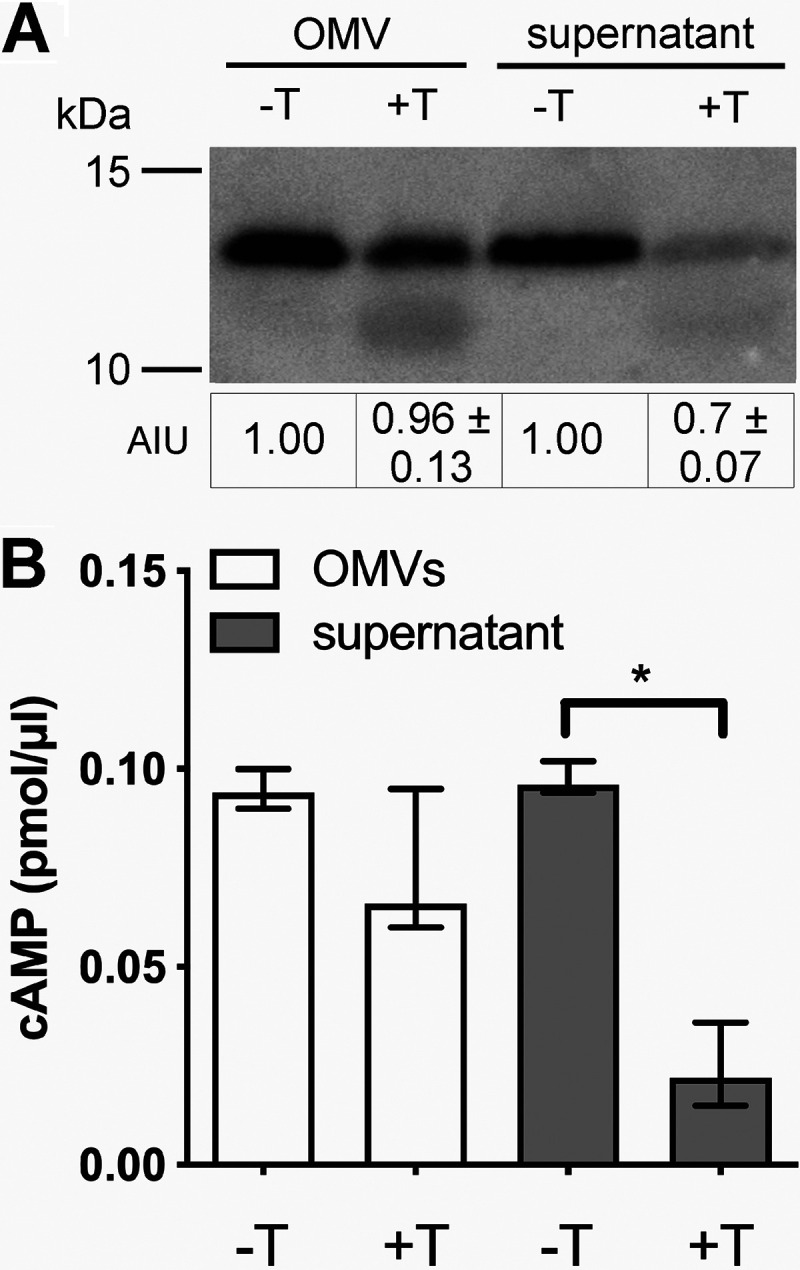
OMV-associated CT is fairly protected from proteolysis by trypsin and remains active. (A) Shown is a representative immunoblot detecting the CT-B subunit in OMVs and supernatant derived from the WT strain after incubation at 37°C for 3 h with (+) or without (−) 3 μg/ml trypsin (T). The commercially available anti-CT antiserum used here detects CT-A and CT-B subunits (see Fig. S5 in the supplemental material). Evaluation was based on the more intense CT-B band, which is most likely due to the one CT-A and five CT-B subunit stoichiometry. Semiquantitative densitometric evaluation of the detected CT-B subunit was performed with Quantity One software (Bio-Rad Laboratories), and results are indicated below the immunoblots as arbitrary intensity units (AIU) (mean AIU with SD [*n* = 5]). Trypsin-digested OMV or supernatant samples were normalized to the respective samples incubated without trypsin, which were set to 1. Silver-stained SDS gels executed in parallel served as loading controls (Fig. S4). (B) The activity of CT in OMVs and supernatant from the WT strain treated with (+) or without (−) 3 μg/ml trypsin (T) was determined by an cAMP assay. HT-29 cells were incubated with the respective samples for 6 h and subsequently processed for the cAMP assay. OMVs and supernatant derived from the Δ*ctx* mutant treated with or without trypsin were also applied to HT-29 cells, but cellular cAMP levels were below the limit of detection (0.0078 pmol/μl) for all Δ*ctx* mutant samples used. Data are presented as median ± IQR (*n* = 5). Asterisks highlight significant differences between respective data sets. *, *P < *0.05, Kruskal-Wallis test followed by *post hoc* Dunn’s multiple comparisons.

To determine the susceptibility of CT to intestinal proteases, WT V. cholerae supernatant and OMVs were incubated with or without trypsin and subsequently subjected to immunoblot analyses. Treatment with trypsin for 3 h reduced the CT in the supernatant derived from WT V. cholerae by approximately 30%, compared to the untreated sample, demonstrating that free CT is susceptible to proteolytic degradation (Fig. 6A). In contrast, OMV-associated CT remained quite stable in the presence of trypsin (Fig. 6A). A silver-stained polyacrylamide gel executed in parallel highlighted the marked reduction of a prominent band at 12 kDa in the supernatant, as well as distinct changes in the OMV protein profile, upon trypsin treatment (Fig. S4). In comparison to the untreated OMV sample, some bands were reduced in intensity or vanished, while two bands below 15 kDa became visible; this suggests that the majority of OMV-associated proteins were protected from trypsin proteolysis (Fig. S4). Finally, supernatant and OMVs treated with or without trypsin were also analyzed for the remaining bioactivity using the cAMP assay in combination with HT-29 cells (Fig. 6B). Upon trypsin digestion, a significant decrease in cAMP levels was observed for the supernatant but not for the OMVs. OMVs and supernatant derived from the Δ*ctx* mutant treated with or without trypsin were applied to HT-29 cells, but basal cAMP levels were below the limit of detection (0.0078 pmol/μl) for all cells treated with Δ*ctx* mutant samples. In summary, this indicates that trypsin can significantly affect the activity of free CT, whereas most OMV-associated CT is protected from proteolysis and remains active even in the presence of trypsin.

10.1128/mBio.00534-21.4 FIG S4 Representative silver-stained gel of OMVs and supernatant (SUP) derived from WT V. cholerae after incubation at 37°C for 3 h with (+) or without (−) 3 μg/ml trypsin (T). SDS-PAGE and subsequent silver staining were executed in parallel with the same samples used for the immunoblot analyses provided in Fig. 6 and serve as a loading control. Molecular mass standards (prestained protein marker, broad range; New England Biolabs) are indicated on the left. Download FIG S4, PDF file, 0.3 MB. Copyright © 2021 Zingl et al. 2021 Zingl et al. https://creativecommons.org/licenses/by/4.0/ This content is distributed under the terms of the Creative Commons Attribution 4.0 International license.

10.1128/mBio.00534-21.5 FIG S5 Detection of CT-A and CT-B subunits by anti-CT antiserum used in this study. Shown is a representative immunoblot detecting CT-A (21.8 kDa) and CT-B (11.6 kDa) subunits in OMVs derived from WT V. cholerae and a Δ*ctx* mutant grown in AKI, as well as purified CT (0.2 μg). Download FIG S5, PDF file, 0.2 MB. Copyright © 2021 Zingl et al. 2021 Zingl et al. https://creativecommons.org/licenses/by/4.0/ This content is distributed under the terms of the Creative Commons Attribution 4.0 International license.

**FIG 7:**
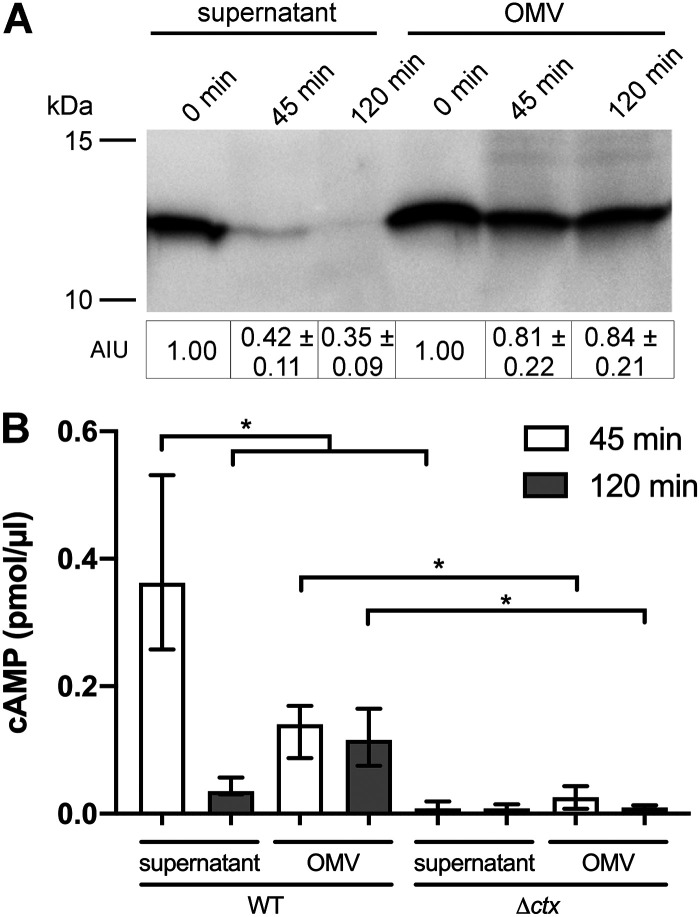
CT associated with OMVs exhibits prolonged stability in the mouse intestine. (A) Shown is a representative immunoblot detecting the CT-B subunit in OMVs and supernatant derived from the WT strain after incubation in mouse intestinal loops for 45 and 120 min. Semiquantitative densitometric evaluation of the detected CT-B-subunit was performed with Quantity One software (Bio-Rad Laboratories) and is indicated below the immunoblots as AIU (mean AIU with SD [*n* = 10]). (B) Activity of CT in OMVs and supernatant from the WT strain in murine intestinal loops was determined by a cAMP assay. Mouse intestinal tissue was ground after exposure to OMVs and supernatant for 45 and 120 min and subsequently processed for the cAMP assay. OMV and supernatant derived from the Δ*ctx* mutant served as controls. Due to the absence of CT in samples derived from the Δ*ctx* mutant, equal OMV amounts or supernatant volumes as used for the WT samples were applied. Data are presented as median ± IQR (*n* = 5). Asterisks highlight significant differences between respective data sets. *, *P < *0.05, Kruskal-Wallis test followed by *post hoc* Dunn’s multiple comparisons.

Next, we wanted to analyze the stability of CT present in the supernatant or associated with OMVs upon exposure to the intestinal milieu. Therefore, we injected supernatant and OMVs derived from WT V. cholerae grown under virulence-inducing conditions into freshly prepared ileal loops for 45 and 120 min (Fig. 7). Subsequent immunoblot analyses of the luminal contents retrieved from the ileal loops revealed differences in CT stability depending on its presence in the supernatant or association with OMVs. In the case of the supernatant-derived CT, a pronounced decrease could be observed already after 45 min (Fig. 7A). In contrast, OMV-associated CT remained quite stable for up to 120 min. Luminal contents from ileal loops injected with supernatant or OMVs derived from the Δ*ctx* mutant did not yield detectable CT bands for the time points analyzed (Fig. S6). In addition, cAMP levels of the ileal loop tissue were determined to assess the bioactivity of the CT present either in the supernatant or associated with OMVs (Fig. 7B). Injection into ileal loops of CT-free OMVs or supernatant derived from the Δ*ctx* mutant served as controls, revealing basal cAMP levels in the tissue (Fig. 7; also see Fig. S6). Intestinal tissue exposed to WT V. cholerae supernatant for 45 min showed relatively high cAMP levels, followed by a significant decrease at 120 min. Thus, at the 45-min time point but not at 120 min, cAMP levels in tissue exposed to WT V. cholerae supernatant were significantly higher than those in tissue exposed to the CT-free supernatant derived from the Δ*ctx* mutant. In contrast, intestinal tissue exposed to WT OMVs showed moderate increases in cAMP levels, compared to the control tissue exposed to Δ*ctx* mutant OMVs. However, elevated cAMP levels upon treatment with WT V. cholerae OMVs remained stable, without a significant decrease at 120 min. Thus, for both time points the cAMP levels in tissue exposed to WT OMVs remained significantly higher than those in tissue exposed to CT-free OMVs derived from the Δ*ctx* mutant. These results suggest that free CT and OMV-associated CT have different stabilities and dynamics of activity in the intestinal milieu. Free CT is highly potent, resulting in a rapid but short rise in cellular cAMP levels. Concordantly, free CT is highly susceptible to intestinal proteolysis. On the other hand, OMV-associated CT exhibits prolonged stability and induces a moderate but sustained increase in cAMP levels.

10.1128/mBio.00534-21.6 FIG S6 Immunoblot of OMVs and supernatant derived from a Δ*ctx* mutant, serving as control samples. Shown is a representative immunoblot to confirm no detectable signal for CT-A (21.8 kDa) or CT-B (11.6 kDa) subunits in OMVs or supernatants derived from the Δ*ctx* mutant after incubation in mouse intestinal loops for 45 and 120 minutes. Purified CT (0.2 μg) served as a control. Download FIG S6, PDF file, 0.5 MB. Copyright © 2021 Zingl et al. 2021 Zingl et al. https://creativecommons.org/licenses/by/4.0/ This content is distributed under the terms of the Creative Commons Attribution 4.0 International license.

## DISCUSSION

It is becoming increasingly evident that OMVs act as vehicles for delivery of bacterial effectors to host cells. Prominent examples are the heat-labile toxin of enterotoxigenic Escherichia coli (42), ClyA of E. coli (43), and the VacA cytotoxin of Helicobacter pylori (44). In the case of V. cholerae, at least three effectors, which are taken up by host cells, have been implicated to be OMV associated (27, 28, 45, 46); these include the VesC protease, which promotes cytotoxic and inflammatory responses, the RTX toxin, which becomes OMV associated in stationary phase and mediates actin polymerization in host cells, and the CT, which was found to be associated with OMVs from classical V. cholerae strains (27, 28, 45, 46). However, information on the molecular principles involving the OMV-associated bacterial effectors required for host cell interactions and internalization, as well as the relevant uptake pathways, was lacking.

In this study, the outer membrane porins, i.e., OmpU and OmpT, were characterized to be the relevant bacterial surface structures required for OMV internalization by intestinal epithelial cells. Inhibitor studies and microscopic analyses indicate that caveolin-mediated endocytosis is the dominant uptake pathway for V. cholerae OMVs. Throughout this study, a V. cholerae O1 El Tor isolate, representing the V. cholerae biotype responsible for all cholera outbreaks of the past 60 years, was used. This allowed us to study the pathophysiological relevance of OMVs from a clinically relevant strain under defined virulence-inducing and non-virulence-inducing conditions, i.e., AKI and LB cultivation. Notably, OMVs derived under virulence-inducing and non-virulence-inducing conditions exhibited similar dependency not only on outer membrane porins but also on the caveolin-mediated endocytosis uptake pathway in both intestinal cell lines tested. This suggests rather conserved internalization mechanisms for host cell uptake of V. cholerae OMVs. It is becoming increasingly evident that several cell surface interactions can contribute to CT uptake into host cells, mainly involving clathrin- and caveolin-mediated endocytosis pathways (47, 48). Internalization of free secreted CT has been historically attributed to the interaction of CT-B with the ganglioside GM1 on the host cell surface (49). However, recent reports highlight that CT-B is also able to bind fucosylated glycoconjugates, e.g., difucosylated blood group antigens, which additionally contribute to CT internalization and host cell intoxication (50–52). Notably, *B4galnt1* knockout mice lacking GM1 still exhibit a robust physiological response to CT, indicating that GM1-independent mechanisms for CT delivery exist *in vivo* (53). In addition to the CT-B interactions with different host cell receptors, the OMV-mediated uptake presented here diversifies the CT delivery routes by the bacterial porins OmpU and OmpT. Thus, V. cholerae may utilize diverse strategies and multiple host receptors to intoxicate target cells with CT.

Throughout the study, OmpU seemed to be the dominant surface structure for cellular uptake of OMVs. Notably, OmpU is the most abundant outer membrane porin on the surface already under non-virulence-inducing conditions, i.e., LB cultivation. Due to activation of the ToxR regulon, OmpU expression is even further facilitated during growth under virulence-inducing conditions, i.e., AKI, while OmpT is repressed. Concordantly, AKI-derived OMVs lacking OmpT are not impaired for cellular internalization. However, a recent study revealed that OmpT is readily depleted from the bacterial surface via OMVs along the *in vivo* adaptation process (4). This surface depletion of OmpT via OMVs upon activation of the ToxR regulon promotes adaptation to bile (4). Thus, OmpT can be present on at least some OMVs derived from AKI cultures. This might explain the residual uptake of OMVs from the Δ*ompU* mutant cultivated in AKI, which is further reduced for OMVs from the Δ*ompU*/*T* double mutant. The slightly more efficient uptake dynamic of AKI-derived OMVs, compared to LB-derived OMVs, seems at least partially independent of the porins, as AKI-derived Δ*ompU*/*T* mutant OMVs show significantly greater uptake than LB-derived Δ*ompU*/*T* mutant OMVs.

In *trans* expression of OmpU or OmpT from an inducible plasmid rescued OMV uptake of an Δ*ompU*/*T* double mutant to similar levels. Thus, dominance by OmpU might be based on stoichiometry and both porins can mediate cellular uptake upon equal expression, an observation that might become relevant in future studies, as the distribution of OmpU and OmpT on OMVs is currently unknown.

OMV association of CT has so far been reported only for the classical biotype (27, 28). Based on the results presented here, OMV association of CT can now be extended to the clinically relevant El Tor isolates. Our data suggest that, of the CT secreted by the V. cholerae El Tor biotype, only about 10% to 15% is associated with OMVs, raising the question of physiological relevance. However, in the intestinal environment, secreted proteinaceous factors like CT might be readily degraded by digestive enzymes of the human host. Indeed, free secreted CT seems to be highly susceptible to trypsin, an abundant serine protease of the human intestinal tract. Trypsin concentrations in human intestinal fluid can be as high as 100 μg/ml (54–56), but V. cholerae penetrates efficiently into the mucosal layer, where trypsin concentrations are likely lower. Thus, a trypsin concentration of 3 μg/ml along with the 3-h incubation period used here seems a reasonable scenario for mucosal colonization of V. cholerae. In contrast to the susceptible free secreted CT in the supernatant, OMV-associated CT exhibits a significantly increased half-life in the presence of trypsin. Prolonged stability of OMV-associated CT is confirmed by *in vivo* assays using murine intestinal loops. Furthermore, the *in vivo* assays suggest that OMV-associated and free CT exhibit different dynamics in cAMP activation. While free CT promotes an intense, short-lived increase of cellular cAMP levels, exposure to OMV-associated CT induces a moderate but sustained elevation of cAMP levels. Thus, OMVs add two new features to their cargo; OMV-associated CT can be taken up via caveolin-mediated endocytosis and withstand the degradative pressure in the intestinal tract. The prolonged half-life of OMV-associated CT may also allow trafficking over longer distances, which could open the possibility that OMV-associated CT is internalized by diverse host cells distant from the primary colonization site.

## MATERIALS AND METHODS

### Bacterial strains and growth conditions.

Bacterial strains and plasmids used in this study are listed in Table S1 in the supplemental material, and oligonucleotides are listed in Table S2. The clinical isolate V. cholerae O1 El Tor SP27 served as the WT strain in all experiments. Unless stated otherwise, all V. cholerae strains were cultivated at 37°C with aeration in LB or under virulence-inducing conditions using AKI according to a standard recipe (57). Escherichia coli strains DH5αλpir and SM10λpir (58) were used for genetic manipulations and grown at 37°C with aeration in LB. Antibiotics and other supplements were used at the following final concentrations: streptomycin (Sm), 100 μg/ml; ampicillin (Ap), 50 μg/ml in combination with other antibiotics; otherwise, 100 μg/ml; kanamycin (Km), 50 μg/ml; sucrose, 10%; arabinose, 0.2%.

10.1128/mBio.00534-21.7 TABLE S1 Strains and plasmids used in this study. Download Table S1, DOCX file, 0.02 MB. Copyright © 2021 Zingl et al. 2021 Zingl et al. https://creativecommons.org/licenses/by/4.0/ This content is distributed under the terms of the Creative Commons Attribution 4.0 International license.

10.1128/mBio.00534-21.8 TABLE S2 Oligonucleotides used in this study. Download Table S2, DOCX file, 0.01 MB. Copyright © 2021 Zingl et al. 2021 Zingl et al. https://creativecommons.org/licenses/by/4.0/ This content is distributed under the terms of the Creative Commons Attribution 4.0 International license.

### Construction of in-frame deletion mutants and expression plasmids.

The isolation of chromosomal DNA, PCRs, the purification of plasmids or PCR products, the construction of suicide and expression plasmids, and the subsequent generation of deletion mutants were carried out as described previously (59, 60). Qiagen plasmid kits were used for isolation of plasmid DNA, and QIAquick gel extraction and QIAquick PCR purification kits (Qiagen) were used for purification of DNA fragments. PCRs for subcloning were carried out using the Q5 high-fidelity DNA polymerase (New England Biolabs), while *Taq* DNA polymerase (New England Biolabs) was used for all other PCRs.

Constructions of a *tcpP* in-frame deletion mutant was carried out as described by Donnenberg and Kaper (61). Briefly, ∼800-bp PCR fragments located upstream and downstream of *tcpP* were amplified using the oligonucleotide pairs TcpP_SacI_1/TcpP_BamHI_2 and TcpP_BamHI_3/TcpP_SacI_4 (Table S2). After digestion of the PCR fragments with the appropriate restriction enzyme (New England Biolabs) (indicated by the name of the oligonucleotide), they were ligated into pCVD442, which was digested with the appropriate restriction enzymes. Unless noted otherwise, ligation products were transformed into DH5αλpir and Ap-resistant (Ap^r^) colonies were characterized for the correct constructs by PCR (and restriction analysis). The corresponding knockout plasmids obtained are listed in Table S1.

To obtain deletion strains, generated derivatives of pCVD442 were transformed into E. coli Sm10λpir and conjugated into V. cholerae. Exconjugants were purified by Sm^r^/Ap^r^ selection. Sucrose selection was used to obtain Ap-sensitive (Ap^s^) colonies, and chromosomal deletions/replacements were confirmed by PCR.

For the construction of the Δ*wav* deletion mutant, a method described by Schild et al. (62) was used, which enabled us to remove a relatively large chromosomal gene cluster. Briefly, the first step was the construction of a *wavL* to *wavI* mutant carrying an insertion of the *kanR* gene (originating from plasmid pACYC177), consisting of about two-thirds of its gene length (KanI) and including the promoter and Shine-Dalgarno sequences. Then, 800-bp-long upstream and downstream sequences of *wavL* were amplified by PCR using oligonucleotide pairs wavL1-SacI/wavL2-BamHI and wavL3-EcoRI/wavL4-XbaI. The 800-bp PCR fragment of KanI was generated using the oligonucleotide pair kanI_BamHI/kanI_EcoRI (62). The three PCR fragments were digested with appropriate restriction enzymes (New England Biolabs) and ligated together into SacI/XbaI-digested pKEK, resulting in the plasmid pKEKwavL::KanI. The plasmid pKEKwavL::KanI was mobilized into V. cholerae via conjugation, yielding the mutant Δ*wavL*::*KanI* after sucrose selection. In the second step, the suicide plasmid pGP704 harboring the downstream sequence of *wavI* and the last two-thirds (C terminal) of the *kanR* gene (KanII) was constructed. The PCR fragments were obtained using the oligonucleotide pair wavI_NcoI/wavI-XbaI for the 800 bp downstream of *wavI* or kanII_SacI/kanII_NcoI for KanII (62), digested with NcoI/Xba or SacI/NcoI, and ligated into SacI/XbaI-digested pGP, resulting in pGPwavIKanII. After conjugation of pGPwavIKanII in Δ*wavL*::*KanI*, we obtained Ap^r^ colonies. Colony purification of some of these cells in the absence of Ap and subsequent selection for Km^r^ cells resulted in Km^r^/Ap^s^ colonies. Deletion of the Km cassette was achieved by pKEK suicide vector mutagenesis using pKEKΔkanR, which was constructed by amplifying PCR fragments using the oligonucleotide pairs wavL1-SacI/wavL2-HindIII and wavI3-HindIII/wavI-XbaI, digested with SacI/HindIII or HindIII/XbaI, and ligated into SacI/XbaI-digested pKEK, resulting in pKEKΔkanR. The correct chromosomal deletion of genes *wavL* to *wavI* was confirmed by PCR to obtain Δ*wavL-wavI*. This step was followed by another deletion of *wavD* to *wavH* conducted in a similar fashion. The 800-bp-long upstream and downstream sequences of *wavD* were amplified by PCR using oligonucleotide pairs wavD1-SacI/wavD2-BamHI and wavD3-EcoRI/wavD4-XbaI 5. The 800-bp PCR fragment of KanI was generated using the oligonucleotide pair kanI_BamHI/kanI_EcoRI (62). The three PCR fragments were digested with appropriate restriction enzymes (New England Biolabs) and ligated together into SacI/XbaI-digested pKEK, resulting in the plasmid pKEKwavD::KanI. The plasmid pKEKwavD::KanI was mobilized into V. cholerae via conjugation, yielding the mutant Δ*wavD*::*KanI* after sucrose selection. In the second step, the suicide plasmid pGP704, harboring the downstream sequence of *wavH* and the last two-thirds (C terminal) of the *kanR* gene (KanII), was constructed. The PCR fragments were obtained using the oligonucleotide pairs wavH-NcoI/wavH-XbaI for the 800 bp downstream of *wavI* and kanII_SacI/kanII_NcoI for KanII (62), digested with NcoI/Xba or SacI/NcoI, and ligated into SacI/XbaI-digested pGP, resulting in pGPwavHKanII. After conjugation of pGPwavHKanII in Δ*wavD*::*KanI*, we obtained Ap^r^ colonies. Colony purification of some of these cells in the absence of Ap and subsequent selection for Km^r^ resulted in Km^r^/Ap^s^ colonies.

### Isolation of OMVs from V. cholerae strains.

OMVs were isolated as described previously (11). Overnight cultures of the respective strains were grown in LB. The respective cultures were diluted (1:100) in LB (non-virulence-inducing conditions) or AKI (virulence-inducing condition). LB and AKI cultures were grown for 4 h anaerobically, followed by 4 h of shaking at 37°C and 180 rpm with aeration. The cells were then removed from the supernatant by centrifugation (9,000 × *g* for 15 min) and subsequent sterile filtration (0.22 μm). The OMVs present in the supernatant were pelleted by subsequent ultracentrifugation (150,000 × *g* at 4°C for 4 h) and resuspended in appropriate volumes of saline to generate a OMV suspension 1,000-fold more concentrated than the original filter-sterilized supernatant. Protein concentrations were determined using the Bradford assay (protein assay dye reagent; Bio-Rad Laboratories) according to the manufacturer's manual and were normalized to the optical density at 600 nm of the respective culture.

### Size measurement.

To estimate the size distributions of the isolated OMVs, dynamic light scattering was carried out using a Zetasizer Nano ZS90 (Malvern, UK). Samples were diluted 1:1,000 in saline and processed at 25°C under standard settings (dispersant refractive index, 1.331; viscosity, 0.89 cP). Three measurements were performed using a measurement angle of 173° (backscatter), automatic measurement duration, and “seek for optimal position” as the positioning setting.

### SDS-PAGE and immunoblot analysis.

Proteins were separated by SDS-PAGE using 15% polyacrylamide gels in combination with the Mini-PROTEAN Tetra cell system (Bio-Rad) (63). As molecular mass standards, prestained protein marker, broad range (New England Biolabs), was used. Subsequently, protein bands were visualized according to the method described by Kang et al. (64) or further processed for immunoblot analysis as described previously (65). Rabbit anti-CT polyclonal antibody (ab51572; Abcam) and horseradish peroxidase (HRP)-linked anti-rabbit IgG were used as primary and secondary antibodies, respectively. Chemiluminescence detection was performed by using the Immun-Star WesternC kit (Bio-Rad Laboratories), with subsequent exposure in a ChemiDoc XRS system (Bio-Rad Laboratories) in combination with Quantity One software (Bio-Rad Laboratories).

### Silver staining.

Silver staining was carried out as described previously (66) using 10 μg of OMVs separated by SDS-PAGE. After electrophoresis, the gel was fixed overnight in 50% methanol with 5% acetic acid in water. After two washing steps for 20 min with 50% methanol in water and another for 20 s with water, the gel was incubated for 1 min in 0.02% sodium thiosulfate. Afterwards, it was washed three times for 20 s in water and incubated for 20 min in the dark in 0.2% silver nitrate solution supplemented with 200 μl/liter formaldehyde. The gel was subsequently washed two times for 20 s in water and submerged in 3% sodium carbonate and 200 μl/liter formaldehyde until developed to satisfaction. The development was stopped with 1% glycerol for 10 min, and the gel was washed with water for 30 min before imaging.

### CT ELISA.

GM1 ELISA was used to quantify the concentration of CT in OMV and cell-free supernatant samples as described previously (67). Equal volumes of supernatant and equal volumes of OMV samples were serially diluted. Different concentrations of purified CT with known concentrations were used as the standards. Ninety-six-well polystyrene microtiter plates were coated with GM1 ganglioside overnight, and 1% (wt/vol) fatty acid-free bovine serum albumin (BSA) was used to block the GM1-coated plates for 1 h at room temperature. Next, 12 μl of crude OMVs and 260 μl of supernatant were added to the wells in duplicate and incubated for 1 h at room temperature. Subsequently, a rabbit anti-CT polyclonal antibody (1:10,000) and then an HRP-linked goat ani-rabbit IgG antibody (1:2,000) were added to the wells and allowed to incubate for 1 h at room temperature each. For development of the CT-antibody complex, tetramethylbenzidine (TMB) substrate solution (Thermo Fisher Scientific) was used according to the manufacturer’s protocol. The color intensity in each well was measured at 485 nm in a plate reader. CT amounts in the samples were estimated by comparison to the standard curve.

### Rhodamine staining of OMVs.

One-milligram protein equivalent of isolated OMVs was diluted in staining buffer (50 mM Na_2_CO_3_, 100 mM NaCl [pH 9.2]) to a final volume of 1 ml. After addition of 100 μl octadecyl rhodamine B chloride (R18) (Thermo Fisher Scientific) to a final concentration of 0.5 mg/ml, OMVs were stained in the dark with constant agitation overnight at room temperature. Finally, R18-labeled OMVs were subjected to density gradient purification as described previously (6). Density gradient-purified R18-labeled OMVs were quantified by the Bradford assay for protein amounts, to allow the use of equal amounts in the uptake assays.

### Cell culture conditions.

HT-29 or Caco-2 cells were grown at 37°C in a CO_2_ incubator in T-175 tissue culture flasks containing Dulbecco's modified Eagle's medium (DMEM)-nutrient F-12 medium (Gibco, USA) supplemented with 10% fetal calf serum (FCS), penicillin G, and glutamate. Cells were seeded in a 96-well plate at a concentration of approximately 1 × 10^5^ cells/well 24 h prior to the internalization assay. For the cAMP assay, HT-29 cells were seeded in 6-well tissue culture plates at a concentration of 6 × 10^5^ cells/well 24 h prior to the assay. Cells were maintained in DMEM-F12 medium supplemented with glutamate and penicillin G but without FCS during the treatment. 3-(4,5-Dimethyl-2-thiazolyl)-2,5-diphenyl-2*H*-tetrazolium bromide (MTT) cell viability assays were routinely performed at the end of the cell culture assays (68, 69), but no significant reduction in metabolic activity could be observed for any condition used in this study (data not shown).

### Uptake assay.

Cellular uptake assays using R18-labeled OMVs were performed as described previously (33, 70). R18-labeled OMVs were diluted in cell culture medium depleted of FCS to a final concentration of 10 ng/μl, based on quantification by the Bradford assay. HT-29 or Caco-2 cells were seeded into black 96-well plates and incubated for 24 h at 37°C. Cells were washed with 200 μl medium depleted of FCS, and 1 μg of protein equivalent of OMVs was added per well. Cells were incubated at 37°C, and fluorescence was measured for 8 h. Commercially available uptake inhibitors (Sigma-Aldrich) were added at the following concentrations: wortmannin, 0.1 μM in DMSO; cytochalasin D, 0.5 μM in DMSO; chlorpromazine, 0.35 μM in double-distilled H_2_O; amiloride, 0.1 mM in DMSO; nystatin, 0.2 μM in DMSO; dynasore, 80 μM in double-distilled H_2_O.

### cAMP assay.

HT-29 cells (6 × 10^5^ cells/well) were incubated for 6 h at 37°C with OMVs or supernatants of the WT strain or the Δ*ompU/T* mutant normalized to equal CT amounts (1 ng), based on ELISA quantification. Due to the absence of CT in samples derived from the Δ*ctx* mutant, equal OMV amounts (based on the Bradford assay) or supernatant volumes, compared to those used for the WT samples, were applied. HT-29 cells were subsequently processed according to the manufacturer’s protocol to quantify the cAMP levels in cellular lysates using the cAMP activity assay kit (Biovision, USA).

### Colocalization studies.

Immunofluorescence microscopy and staining were adapted from previous publications with slight modifications (38). Briefly, Caco-2 cells (1 × 10^5^ cells/well) were incubated for 10 min at 37°C with WT OMVs (0.1 mg/ml, based on the Bradford assay) or solvent control (saline) in 8-well slides (Dibco). After removal of the OMVs or saline solution, the cells were fixed with 3.7% paraformaldehyde and quenched with 0.2 M glycine. For permeabilization, cells were treated with 1% saponin in phosphate-buffered saline (PBS), followed by a blocking step with 5% goat serum in PBS. Rabbit anti-caveolin 1 (PA1-064; Invitrogen) (1:200 in PBS with 0.1% saponin and 1% goat serum) and mouse anti-OMV (1:100 in PBS with 0.1% saponin and 1% goat serum [71]) were used as primary antibodies, and fluorescein isothiocyanate (FITC)-conjugated goat anti-mouse IgG (ab6717; Abcam) (1:1,000 in PBS with 0.1% saponin and 1% goat serum) and Cy3-conjugated goat anti-rabbit IgG (111-165-144; Jackson Immunoresearch) (1:1,000 in PBS with 0.1% saponin and 1% goat serum) were used as secondary antibodies. Subsequently, cells were stained with 4′,6-diamidino-2-phenylindole (DAPI) (1 μg/ml in PBS). Images were recorded using an inverted microscope (Eclipse Ti-E; Nikon) equipped with a Nikon DS-Qi2 camera and a Nikon CFI Plan Apo Lambda 60× oil objective (numerical aperture, 1.40). DAPI was detected using excitation at 340 to 380 nm and emission at 435 to 485 nm, FITC was detected using excitation at 465 to 495 nm and emission at 515 to 555 nm, and Cy3 was detected using excitation at 528 to 553 nm and emission at 590 to 650 nm. All images were analyzed by Nis-Elements BR version 4.30.02 software.

### Trypsin susceptibility assay.

Proteolytic trypsin digestion of OMVs and supernatants was performed to analyze the susceptibility of OMV-associated and secreted CT to proteolysis. Trypsin was reconstituted in 100 mM Tris-HCl (pH 7.8) with 10 mM calcium chloride. OMVs and supernatants first were normalized to equal CT amounts based on ELISA quantification and subsequently were treated for 3 h at 37°C with equal volumes of trypsin (3 μg/ml diluted in reaction buffer [50 mM ammonium bicarbonate]) or reaction buffer only. Equal volumes of the samples were analyzed by SDS-PAGE and immunoblot analysis using the anti-CT antibody (ab51572; Abcam).

### Ethics statement.

Adult C57/BL6 mice (at least 8 weeks of age; Janvier) were used in all experiments, in accordance with the rules of the ethics committee at the University of Graz and the corresponding animal protocol, which was approved by the federal ministry BMBWF (protocol 39/12/75ex2017/18). Mice were housed with food and water available *ad libitum* and monitored under the care of full-time staff members.

### Stability and activity of CT in intestinal loops.

Mice were fasted for 24 h and euthanized by cervical dislocation before the intestinal tract was removed and immediately placed in cell culture medium (DMEM-F12 medium; Gibco, USA) preheated to 37°C. For each intestine, two ileal loops of approximately 2-cm length were separated by suture, with at least 1-cm distance between the loops. The closed ileal loops were injected with approximately 300 μl of solution containing either OMVs or supernatant and were incubated at 37°C in 5% CO_2_. While the supernatant was used directly, OMVs were adjusted to equal CT amounts, compared with the supernatant, based on ELISA quantification. Due to the absence of CT in samples derived from the Δ*ctx* mutant, equal supernatant volumes or OMV amounts (based on the Bradford assay), compared with those used for the WT samples, were applied.

At the specified time points after inoculation (45 and 120 min), luminal contents of the ileal loops were released in a cryotube containing glass beads (0.1 mm in diameter; Roth) by opening of the loop with surgical scissors. Luminal contents were homogenized using the PowerLyser benchtop bead-based homogenizer (MO BIO Laboratories, Inc.) for 1 min at 3,400 rpm and were subjected to immunoblot analyses using the anti-CT antibody (ab51572; Abcam). The initial inocula (time of  0 min) were included in immunoblot analyses to confirm the presence of equal CT amounts.

The remaining tissue (approximately 0.5 g) was transferred to a cryotube containing 200 μl HCl (0.1 M). Glass beads (0.5 mm in diameter; Roth) were added to the sample, and the sample was lysed using the PowerLyser benchtop bead-based homogenizer (MO BIO Laboratories, Inc.) for 1 min at 3,400 rpm. Samples were centrifuged for 5 min at 5,000 × *g* to remove intestinal debris and glass beads. The supernatant was used for cAMP quantification by ELISA (see above).
