# Supplementary material for: Outer Membrane Vesicles of Vibrio cholerae Protect and Deliver Active Cholera Toxin to Host Cells via Porin-Dependent Uptake
Source: mBio. 2021 May 26;12(3):e00534-21. doi: 10.1128/mBio.00534-21 (PMC8262896; doi:10.1128/mBio.00534-21)
Supplement: Fig S1 [file mbio.00534-21-sf001.pdf]

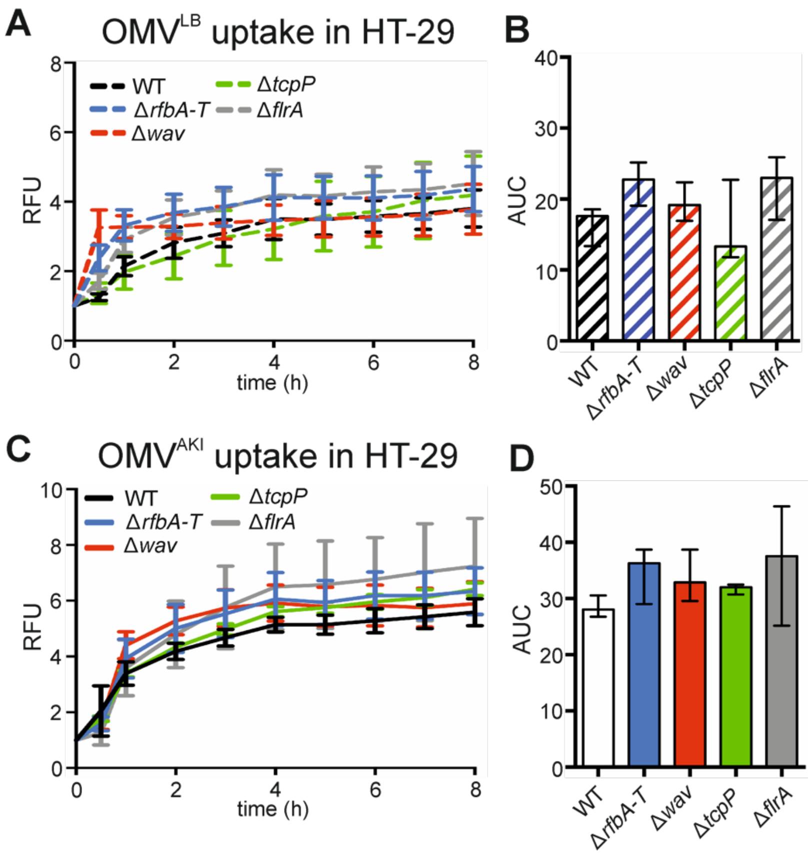

Figure S1: Uptake of OMVs derived from *V. cholerae* WT and diverse surface mutants by intestinal epithelial cells. (A and C) HT-29 intestinal cells were incubated for 8 h with rhodamine-labeled OMVs derived from *V. cholerae* WT as well as Δ*rfbA-rfbT*, Δ*wav*, Δ*tcpP* and Δ*flrA* mutants grown in LB (A) or AKI (C). Uptake is detected by an increase in relative fluorescence units (RFU) measured every hour. Wells containing rhodamine-labeled OMVs without cells served as a blank. Shown is the mean ± SD, n ≥ 8. **(B and D)** Shown are the median area under the curve (AUC) values ± IQR retrieved from the uptake analyses of LB (B) or AKI (D) values.
