# Supplementary material for: Outer Membrane Vesicles of Vibrio cholerae Protect and Deliver Active Cholera Toxin to Host Cells via Porin-Dependent Uptake
Source: mBio. 2021 May 26;12(3):e00534-21. doi: 10.1128/mBio.00534-21 (PMC8262896; doi:10.1128/mBio.00534-21)
Supplement: FIG S2 [file mbio.00534-21-sf002.pdf]

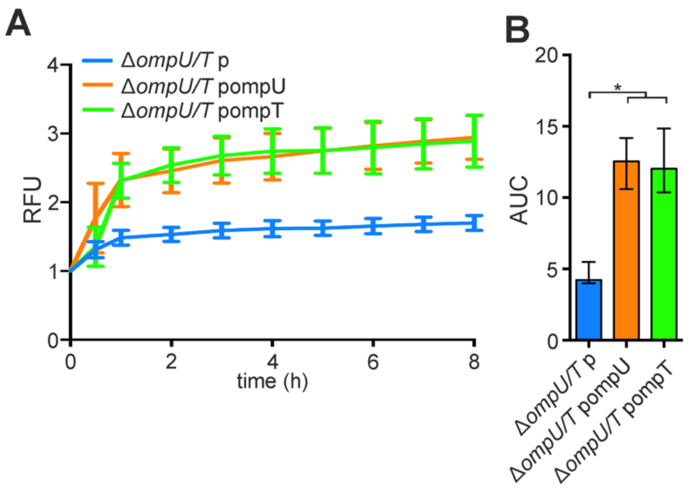

Figure S2: Expression of OmpU and OmpT in trans restores OMV uptake in intestinal epithelial cells. (A) HT-29 intestinal cells were incubated for 8 h with rhodamine-labeled OMVs derived from *V. cholerae* Δ*ompU*/*T* p, Δ*ompU*/*T* pOmpU and Δ*ompU*/*T* pOmpT grown in AKI. Uptake is detected by an increase in relative fluorescence units (RFU) measured every hour. Wells containing rhodamine-labeled OMVs without cells served as a blank. Shown is the mean ± SD, n ≥ 8. **(B)** Shown are the median area under the curve (AUC) values ± IQR retrieved from the uptake analyses presented in panel A. Asterisks highlight significant differences between respective data sets (* $P < 0.05$ Kruskal-Wallis test followed by *post hoc* Dunn's multiple comparisons).
