# Supplementary material for: Outer Membrane Vesicles of Vibrio cholerae Protect and Deliver Active Cholera Toxin to Host Cells via Porin-Dependent Uptake
Source: mBio. 2021 May 26;12(3):e00534-21. doi: 10.1128/mBio.00534-21 (PMC8262896; doi:10.1128/mBio.00534-21)
Supplement: FIG S3 [file mbio.00534-21-sf003.pdf]

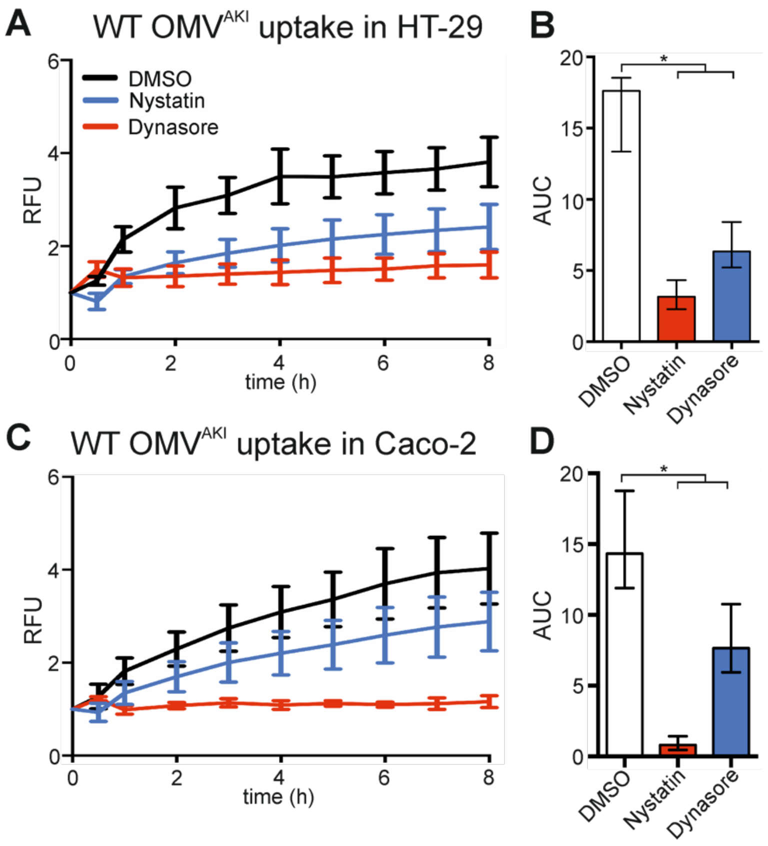

Figure S3: Dynasore or nystatin also reduce uptake of OMVs derived from *V. cholerae* WT grown in LB. (A and C) Intestinal epithelial cells HT-29 (A) or Caco-2 (C) were incubated for 8 h with rhodamine-labeled OMVs derived from WT in presence of uptake inhibitors nystatin, dynasore or the solvent DMSO (control). Uptake is detected by an increase in relative fluorescence units (RFU) measured every hour. Wells containing rhodamine-labeled OMV from WT without cells served as a blank. Shown is the mean ± SD ($n \geq 8$). **(B and D)** Shown are the median area under the curve (AUC) values ± IQR retrieved from the uptake analyses in HT29 (A) and Caco-2 (C), respectively. Asterisks highlight significant differences between respective data sets (* $P < 0.05$ Kruskal-Wallis test followed by *post hoc* Dunn's multiple comparisons).
