# Supplementary material for: Outer Membrane Vesicles of Vibrio cholerae Protect and Deliver Active Cholera Toxin to Host Cells via Porin-Dependent Uptake
Source: mBio. 2021 May 26;12(3):e00534-21. doi: 10.1128/mBio.00534-21 (PMC8262896; doi:10.1128/mBio.00534-21)
Supplement: FIG S4 [file mbio.00534-21-sf004.pdf]

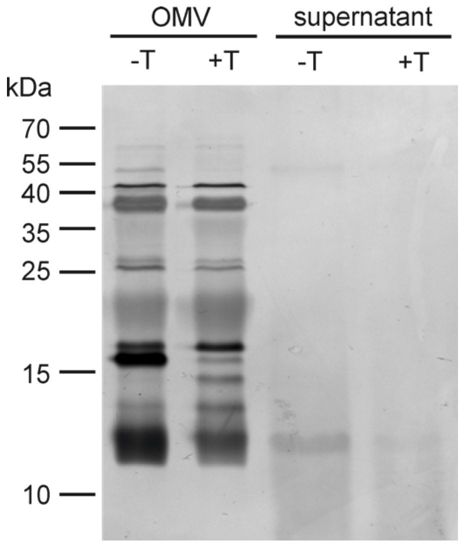

Figure S4: Representative silver-stained gel of OMVs and supernatant (SUP) derived from WT after incubation at 37ºC for 3 hours with (+) or without (-) 3 µg/ml trypsin (T). SDS-PAGE and subsequent silver staining was executed in parallel with the same samples used for the immunoblot analyses provided in Fig. 6 and serves as a loading control. Molecular mass standards (Prestained Protein Marker Broad Range - New England Biolab) are indicated on the left.
