# Supplementary material for: Outer Membrane Vesicles of Vibrio cholerae Protect and Deliver Active Cholera Toxin to Host Cells via Porin-Dependent Uptake
Source: mBio. 2021 May 26;12(3):e00534-21. doi: 10.1128/mBio.00534-21 (PMC8262896; doi:10.1128/mBio.00534-21)
Supplement: FIG S5 [file mbio.00534-21-sf005.pdf]

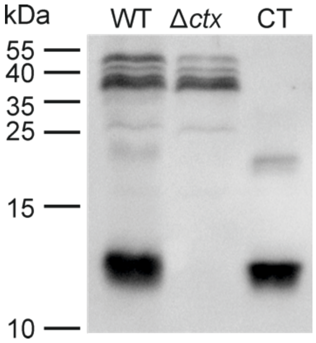

Figure S5: Detection of CT-A and CT-B subunits by anti-CT antiserum used in this study. Shown is a representative immunoblot detecting CT-A (21.8 kDa) and CT-B (11.6 kDa) subunits in OMVs derived from *V. cholerae* WT and a Δ*ctx* mutant grown in AKI as well as purified CT (0.2 µg).
