# Supplementary material for: Outer Membrane Vesicles of Vibrio cholerae Protect and Deliver Active Cholera Toxin to Host Cells via Porin-Dependent Uptake
Source: mBio. 2021 May 26;12(3):e00534-21. doi: 10.1128/mBio.00534-21 (PMC8262896; doi:10.1128/mBio.00534-21)
Supplement: FIG S6 [file mbio.00534-21-sf006.pdf]

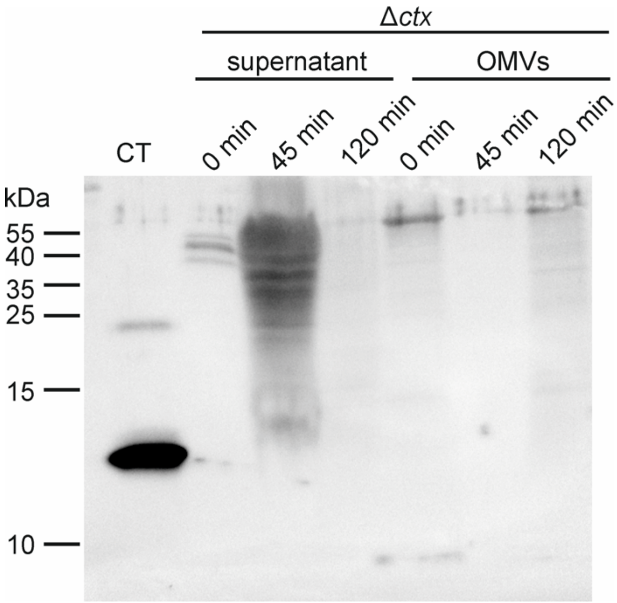

Figure S6: Immunoblot of OMVs and supernatant derived from a Δ*ctx* mutant serving as control samples. Shown is a representative immunoblot to confirm no detectable signal for CT-A (21.8 kDa) or CT-B (11.6 kDa) subunits in OMVs or supernatants derived from the Δ*ctx* mutant after incubation in mouse intestinal loops for 45 and 120 minutes. Purified CT (0.2 µg) served as control.
