# Supplementary material for: Outer Membrane Vesicles of Vibrio cholerae Protect and Deliver Active Cholera Toxin to Host Cells via Porin-Dependent Uptake
Source: mBio. 2021 May 26;12(3):e00534-21. doi: 10.1128/mBio.00534-21 (PMC8262896; doi:10.1128/mBio.00534-21)
Supplement: TABLE S1 [file mbio.00534-21-st001.docx]

**Table S1. Strains and plasmids used in this study.**

| **bacterial strains** | **description** | **reference^1^** |
| --- | --- | --- |
| DH5αλpir | F^-^ *endA1 glnV44 thi-1 recA1 relA1 gyrA96 deoR nupG* Φ80d*lacZ*ΔM15 Δ(*lacZYA-argF*) U169 *hsdR17*(r_K_^-^ m_K_^+^) λ*pir*RK6 | (1) |
| SM10λpir | *thi thr leu tonA lacY supE recA::*RPA-2-Te::Mu λ*pir*, Km^R^ | (1) |
| WT | P27459-S, wild type *V. cholerae* strain serogroup, O1; biotype, El Tor; serotype, Inaba; spontaneous Sm^R^ mutant of E7946; clinical isolate from Bahrain 1978; *hapR*^+^, Sm^R^ | (2) |
| Δ*ctx* | Deletion of *ctxAB* in WT, Sm^R^ | This paper |
| Δ*ompU* | Deletion of *ompU* in WT, Sm^R^ | This paper |
| Δ*ompT* | Deletion of *ompT* in WT, Sm^R^ | This paper |
| Δ*ompU/T* | Deletion of *ompU* and *ompT* in WT, Sm^R^ | This paper |
| Δ*toxR* | Deletion of *toxR* in WT, Sm^R^ | (3) |
| Δ*rfb* | Deletion of *rfbA-T* in WT, Sm^R^ | (4) |
| Δ*wavL*::*KanI* | Kan fragment inserted into wavL in WT, Sm^R^ | This paper |
| Δ*wavD*::*KanI* | Kan fragment inserted into wavI in Δ*wavL-I*, Sm^R^ | This paper |
| Δ*wavL-I* | Deletion of *wavL-I in* WT, Sm^R^, Km^R^ | This paper |
| Δ*wav* | Deletion of *wav* in WT, Sm^R^ | This paper |
| Δ*tcpP* | Deletion of *tcpT* in WT, Sm^R^ | This paper |
| Δ*flrA* | Deletion of *flrA* in WT, Sm^R^ | (5) |
|  |  |  |
| **plasmids** | | |
| pCVD442 | *ori6K mobRP4 sacB*, Ap^R^ | (6) |
| pCVDΔompU | pCVD442 with up- and downstream fragments of *ompU*, Ap^R^ | (7) |
| pCVDΔompT | pCVD442 with up- and downstream fragments of *ompT*, Ap^R^ | (7) |
| pCVDΔtcpP | pCVD442 with up- and downstream fragments of *tcpP*, Ap^R^ | This paper |
| pCVDΔflrA | pCVD442 with up- and downstream fragments of *flrA*, Ap^R^ | (8) |
| pCVDΔctxAB | pCVD442 with up- and downstream fragments of RS1-CTX phage-TLC, Ap^R^ | (9) |
| pKEKwavL::KanI | ∆*wavL*::KanI from P27459 in pKEK229, Ap^R^ | This paper |
| pKEKwavD::KanI | ∆*wavD*::KanI from P27459 in pKEK229, Ap^R^ | This paper |
| pGPwavIKanII | ’wavI’ von P27459 and KanII in pGP704, Ap^R^ | This paper |
| pGPwavHKanII | ’wavH’ von P27459 and KanII in pGP704, Ap^R^ | This paper |
| pKEK∆kanR | ‘wavI’ und ‘wavL’ from P27459 in pKEK229, Ap^R^ | This paper |
| p | pBAD30, IncQ broad-host-range low-copy-number cloning vector, Arabinose-inducible, Ap^r^ | (10) |
| pOmpU | Expression plasmid with *ompU* in pBAD30, Ap^r^ | (11) |
| pOmpT | Expression plasmid with *ompT* in pBAD30, Ap^r^ | (11) |

**^1^References**

1. Kolter R, Inuzuka M, Helinski DR. 1978. Trans-complementation-dependent replication of a low molecular weight origin fragment from plasmid R6K. Cell 15:1199-1208.

2. Nesper J, Kapfhammer D, Klose KE, Merkert H, Reidl J. 2000. Characterization of *Vibrio cholerae* O1 antigen as the bacteriophage K139 receptor and identification of IS1004 insertions aborting O1 antigen biosynthesis. J Bacteriol 182:5097-104.

3. Fengler VH, Boritsch EC, Tutz S, Seper A, Ebner H, Roier S, Schild S, Reidl J. 2012. Disulfide bond formation and ToxR activity in *Vibrio cholerae*. PLoS One 7:e47756.

4. Schild S, Lamprecht AK, Fourestier C, Lauriano CM, Klose KE, Reidl J. 2005. Characterizing lipopolysaccharide and core lipid A mutant O1 and O139 *Vibrio cholerae* strains for adherence properties on mucus-producing cell line HT29-Rev MTX and virulence in mice. Int J Med Microbiol 295:243-51.

5. Butler SM, Camilli A. 2005. Going against the grain: chemotaxis and infection in *Vibrio cholerae*. Nat Rev Microbiol 3:611-20.

6. Donnenberg MS, Kaper JB. 1991. Construction of an *eae* deletion mutant of enteropathogenic *Escherichia coli* by using a positive-selection suicide vector. Infect Immun 59:4310-4317.

7. Osorio CG, Martinez-Wilson H, Camilli A. 2004. The ompU Paralogue vca1008 is required for virulence of *Vibrio cholerae*. J Bacteriol 186:5167-71.

8. Moisi M, Jenul C, Butler SM, New A, Tutz S, Reidl J, Klose KE, Camilli A, Schild S. 2009. A novel regulatory protein involved in motility of *Vibrio cholerae*. J Bacteriol 191:7027-38.

9. Bishop AL, Schild S, Patimalla B, Klein B, Camilli A. 2010. Mucosal immunization with *Vibrio cholerae* outer membrane vesicles provides maternal protection mediated by antilipopolysaccharide antibodies that inhibit bacterial motility. Infect Immun 78:4402-4420.

10. Guzman L-M, Beblin D, Carson MJ, Beckwith J. 1995. Tight regulation, modulation, and high-level expression by vectors containing the arabinose pBAD promotor. J Bacteriol 177:4121-4130.

11. Li CC, Crawford JA, DiRita VJ, Kaper JB. 2000. Molecular cloning and transcriptional regulation of ompT, a ToxR-repressed gene in *Vibrio cholerae*. Mol Microbiol 35:189-203.
