# Supplementary material for: Outer Membrane Vesicles of Vibrio cholerae Protect and Deliver Active Cholera Toxin to Host Cells via Porin-Dependent Uptake
Source: mBio. 2021 May 26;12(3):e00534-21. doi: 10.1128/mBio.00534-21 (PMC8262896; doi:10.1128/mBio.00534-21)
Supplement: TABLE S2 [file mbio.00534-21-st002.docx]

**Table S2. Oligonucleotides used in this study.**

| **Oligonucleotides** | | |
| --- | --- | --- |
| TcpP_SacI_1 | AAAGAGCTCCTGTAAACATGGTGCCATCTA^1^ | This paper |
| TcpP_BamHI_2 | AAAGGATCCTACTTTACATTTTCTTAATAA | This paper |
| TcpP_BamHI_3 | AAAGGATCCCAACATCAGTGTTCCGTGAA | This paper |
| TcpP_SacI_4 | AAATCTAGACGGAGTGGAGATCGAAATTAT | This paper |
| kanI-BamHI | ATGGATCCTTCAACTCAGCAAAAGT | This paper |
| kanI-EcoRI | TAGAATTCCGACTCGTCCAACATCAATA | This paper |
| wavL1-SacI | ATGAGCTCACTCGCAGTGG | This paper |
| wavL2-BamHI | AAGGATCCACTGAGTCGGCCAATGATA | This paper |
| wavL3-EcoRI | TAGAATTCCAGCAAATCCCCGCTTT | This paper |
| wavL4-XbaI | AATCTAGAGTCATGTAACGCTTTAACTT | This paper |
| kanII-SacI | TAGAGCTCATGGATGCTGATTTATATGGGT | This paper |
| kanII-NcoI | TCCCATGGTCAGCGTAATGCTCTGCCAGT | This paper |
| wavI-NcoI | TTCCATGGGGGTGTATCACT | This paper |
| wavI-XbaI | AATCTAGAGGTTTCGCCATACTGTTAA | This paper |
| wavL2-HindIII | TTAAGCTTACTGAGTCGGCCAATGATA | This paper |
| wavI3-HindIII | TTAAGCTTCCATGGGGGTGTATCACT | This paper |
| wavI4-XbaI | AATCTAGAGGTTTCGCCATACTGTTAA | This paper |
| wavD1-SacI | ATGAGCTCTAGCTATAAACATGAAGAGTAAA | This paper |
| wavD2-BamHI | ATGGATCCGCGAAATAATTAGAACAAACAAGCCT | This paper |
| wavD3-EcoRI | TTGAATTCTTCTTCTCTGATTTTTTCCAATTT | This paper |
| wavD4-XbaI | TTTCTAGAAGTTTAATGGAGTGTTTTTAGTTTT | This paper |
| wavH-NcoI | AACCATGGATCTCTTCTAAATTCATCGAGGCA | This paper |
| wavH-XbaI | TTTCTAGATATCACCCACGCACTCATCA | This paper |

^1^ restriction sites are underlined
